# Altered contractility in mutation-specific hypertrophic cardiomyopathy: A mechano-energetic *in silico* study with pharmacological insights

**DOI:** 10.3389/fphys.2022.1010786

**Published:** 2022-10-31

**Authors:** Mohamadamin Forouzandehmehr, Michelangelo Paci, Jussi T Koivumäki, Jari Hyttinen

**Affiliations:** Faculty of Medicine and Health Technology, Tampere University, Tampere, Finland

**Keywords:** in silico modeling, human stem cell-derived cardiomyocyte, action potential, immature cardiomyocytes, cardiac metabolism, hypertrophic cardiomyopathy, pharmacology

## Abstract

**Introduction:** Mavacamten (MAVA), Blebbistatin (BLEB), and Omecamtiv mecarbil (OM) are promising drugs directly targeting sarcomere dynamics, with demonstrated efficacy against hypertrophic cardiomyopathy (HCM) in (pre)clinical trials. However, the molecular mechanism affecting cardiac contractility regulation, and the diseased cell mechano-energetics are not fully understood yet.

**Methods:** We present a new metabolite-sensitive computational model of human induced pluripotent stem cell-derived cardiomyocytes (hiPSC-CMs) electromechanics to investigate the pathology of R403Q HCM mutation and the effect of MAVA, BLEB, and OM on the cell mechano-energetics.

**Results:** We offer a mechano-energetic HCM calibration of the model, capturing the prolonged contractile relaxation due to R403Q mutation (∼33%), without assuming any further modifications such as an additional Ca^2+^ flux to the thin filaments. The HCM model variant correctly predicts the negligible alteration in ATPase activity in R403Q HCM condition compared to normal hiPSC-CMs. The simulated inotropic effects of MAVA, OM, and BLEB, along with the ATPase activities in the control and HCM model variant agree with *in vitro* results from different labs. The proposed model recapitulates the tension-Ca^2+^ relationship and action potential duration change due to 1 µM OM and 5 µM BLEB, consistently with *in vitro* data. Finally, our model replicates the experimental dose-dependent effect of OM and BLEB on the normalized isometric tension.

**Conclusion:** This work is a step toward deep-phenotyping the mutation-specific HCM pathophysiology, manifesting as altered interfilament kinetics. Accordingly, the modeling efforts lend original insights into the MAVA, BLEB, and OM contributions to a new interfilament balance resulting in a cardioprotective effect.

## Introduction

With a prevalence ranging from of 1/500 to 1/200 (Semsarian et al., 2015; Malhotra and Sharma, 2017), HCM represents the most prevalent genetic cardiac disorder mainly associated with pathogenic variants in sarcomere protein genes (Santini et al., 2020). Pathologies such as myocardium hypercontractility (Sarkar et al., 2020), impaired relaxation (Toepfer et al., 2020), elevated cardiac energy consumption, diastolic dysfunction, arrhythmogenesis, and heart failure (Sarkar et al., 2020) manifest due to such variants. The driver of the cyclic interactions between thin and thick filaments is the ATP hydrolysis by myosin-the enzymatic motor of sarcomere (Spudich, 2014). In HCM, myosin binding protein C and adult cardiac myosin isoforms (primarily encoded by MYBPS3 and MYH7 genes, respectively) host most of these pathogenic variants in sarcomere (Ho et al., 2018; Toepfer et al., 2019; Schmid and Toepfer, 2021).

Mavacamten (MAVA), Blebbistatin (BLEB), and Omecamtiv mecarbil (OM) are compounds directly modulating myofilament dynamics with promising effectiveness in treatment of sarcomeric cardiomyopathies. MAVA is an allosteric inhibitor of cardiac myosin ATPase with a negative inotropic effect and demonstrating efficacy in R403Q HCM clinical trials (Green et al., 2016; Santini et al., 2020). BLEB, a well characterized ATPase inhibitor, alters the Ca^2+^ sensitivity of the myofilament and has been widely used in trials (Kampourakis et al., 2018; Rahman et al., 2018; Wang et al., 2018; Gyimesi et al., 2021). OM is a recently developed myosin ATPase activator with a positive inotropic effect enhancing cardiac contractility (Tsukamoto, 2019).

The mechanisms of action of these drugs and their effects on cardiomyocyte electro-mechano-energetics are still to be fully known and under active research (Tsukamoto, 2019; Santini et al., 2020). Accordingly, computational studies on the effects of these drugs are mostly lacking. Focusing on R403Q HCM mutation, Margara et al. (2021) investigated the efficacy of MAVA, simulating the tension-Ca^2+^ relationship and active tension curves. They hypothesized that the impaired tension relaxation phase is caused by feedback from crossbridge (XB) cycling to the thin filament. Although this assumption results in consistent simulated impaired tension relaxation, the proposed MAVA mechanism of action coupled with a lack of a metabolite-sensitive mechanism in the contractile element (CE) urged us to investigate further the cause of this impairment. On the other hand, *in silico* models have been employed to probe the effect of BLEB beside animal muscle fibre experiments (Rahman et al., 2018), highlighting the role of BLEB in shifting the rate-limiting momentum from weakly to strongly bound states in XB cycling (Rahman et al., 2018). Finally, as an ion channel study, the effect of OM has been simulated using an *in silico* model of human ventricular action potential (AP) focusing on pro-arrhythmic assessments (Qu et al., 2021). Of note, Qu et al. report an IC_50_ of 125.5 µM highlighting the significance of OM influence on the CE in 1–10 µM of OM compared with channel blocking formalism (Qu et al., 2021). In summary, to the best of our knowledge, no computational study has reported experimentally validated drug-induced Ca^2+^ sensitivity, ATPase dynamics, and dose-dependent effect of MAVA, BLEB, and OM on the tension-Ca^2+^ relationship, regarding the HCM pathophysiology. This coupled with the facts that these compounds are essentially myosin ATPase activators/inhibitors necessitate studying the drug effects using advanced metabolite-sensitive models. In addition, HCM mutations misregulate sarcomere function and cardiac energy consumption (van der Velden et al., 2018). This further highlights the need for a model that enables capturing the electro-mechano-energetics in pathophysiological investigations.

**FIGURE 1:**
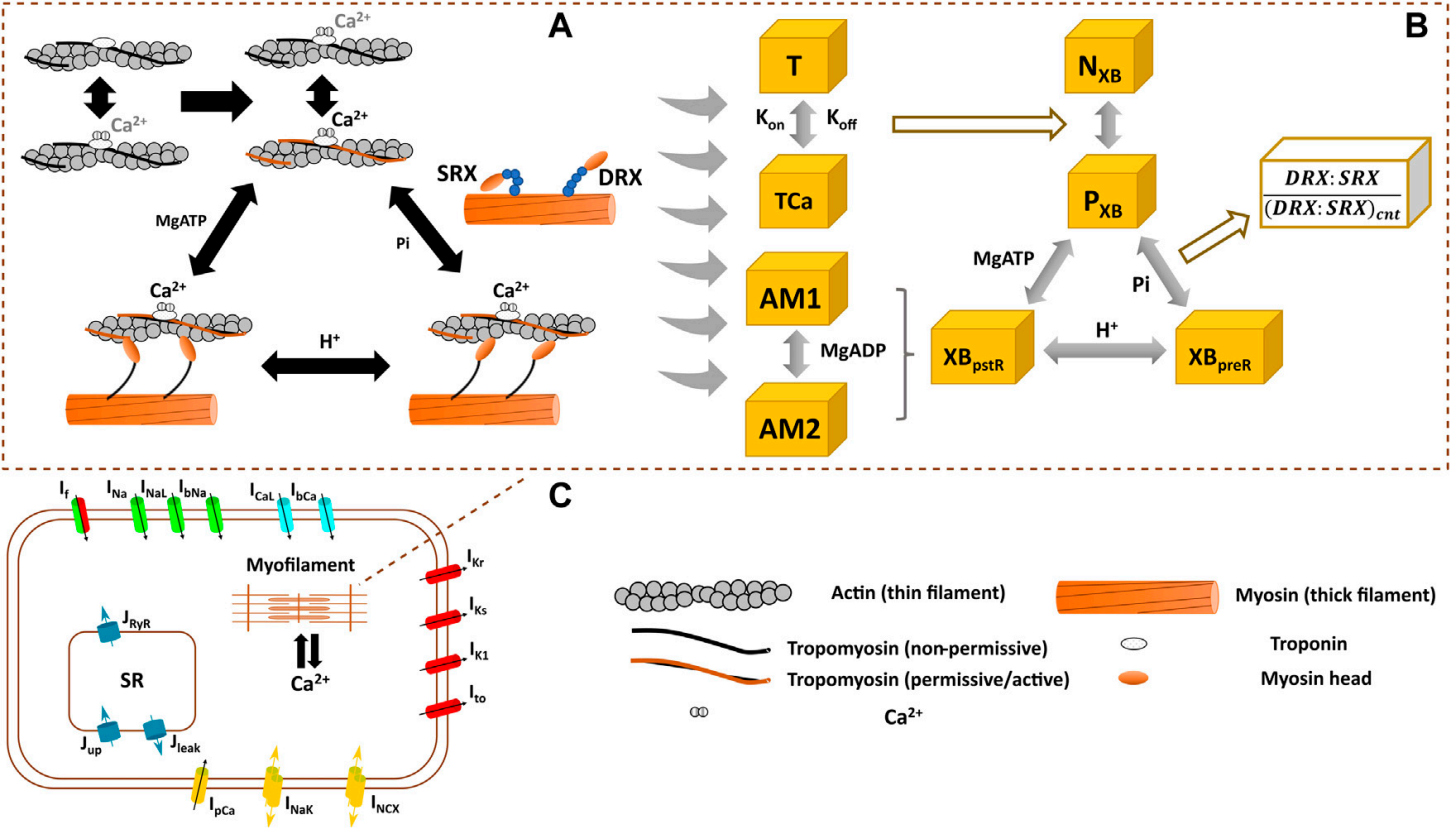
Schematics of the interfilament coupling in cardiac force generation **(A)** the modelled crossbridge (XB) cycling used in hiMCE model **(B)**, and the schematic of hiPSC-CM cell main functional components **(C)**. DRX: Disturbed relax state. SRX: Super relaxed state. T: troponin, TCa: Ca^2+^ bound troponin, N_XB_: non-permissive state preventing XB formation, P_XB_: permissive state of XB formation, XB_preR_: strongly bound XB before isomerised rotation, XB_pstR_: XB in strongly bound post isomerised rotation state, AM1 and AM2 are strongly-bound rapid equilibrium substates contributing equally to the force generation and we assumed MgADP binds to AM1 (Tran et al., 2010).

Here, we investigate the pathophysiology of HCM R403Q myosin mutation using a computational metabolite-sensitive model of hiPSC-CMs electromechanics developed based on ordinary differential equations (ODEs). This model, named hiMCE, is an update of our previous model of hiPSC-CM electromechanics (Forouzandehmehr et al., 2021) capturing ATPase activity and accounting for metabolite-sensitive kinetics (Figure 1) in the XB cycling and extending the capacity of models of the molecular mechanism of contraction and the drug effect predictions. The drugs studied here are modulators of myofilament dynamics, we reparametrized the CE of the model using available experimental data and presented novel mechanistic methods to simulate the effect of MAVA, OM, and BLEB on the Ca^2+^ sensitivity, contractility, and energetics of the hiPSC-CMs.

## Methods

### Extension to a metabolite-sensitive contractile element

We previously integrated a reparametrized mathematical model of the CE by Rice et al. (2008) with a new passive force handling into the hiPSC-CM model of electrophysiology by Paci et al. (2020) and studied the inotropic effect of different compounds (Forouzandehmehr et al., 2021). Based on (Rice et al. (2008) CE, Tran et al. (2017) introduced a mathematical model of a CE that incorporated metabolic-sensitivity. This was achieved by extending the model by Rice et al. (2008), with new parameters that account for the competitive binding of metabolic protons (H^+^) to the binding sites of Ca^2+^ on troponin C, and incorporates the binding kinetics of MgADP in the XB cycling (Figure 1). The extended mechanistic description divides the strongly bound state post isomerized rotation (XB_pstR_) into two substates in rapid equilibrium, AM1 and AM2, to capture MgADP binding kinetics in the XBs (Figure 1).

The thermodynamically constrained model of XB kinetics and Ca^2+^ activation is divided into four states including a non-permissive (N_XB_), a permissive (P_XB_), a pre power stroke state (XB_preR_), and a post power stroke state (XB_pstR_) (Figure 1) (Tran et al., 2017). XB_preR_ and XB_pstR_ states both denote the state of strongly bound myosin heads to the actins (Rice et al., 2008; Tran et al., 2017). In diastole, XBs are in N_XB_ state and when activated by Ca^2+^ XBs enter P_XB_ state where they can participate in the binding and unbinding of myosin heads and processes of tension generation (Figure 1) (Tran et al., 2017). The active tension produced as the result of the process is equal to the product of myosin head strain and strongly bound states fractional occupancy (Tran et al., 2017).

In the present work, we have taken this metabolite-sensitive CE model proposed by Tran et al. (2017), and modified it to incorporate active contraction mechanisms integrated with the Paci2020 model of hiPSC-CMs electrophysiology (Paci et al., 2020). This extended CE model was manually tuned using the information from a previous sensitivity analysis of the contractile machinery (Forouzandehmehr et al., 2021) (Table 1). We calibrated the model to capture the AP, CaT, and contractile experimental biomarkers measured in hiPSC-CMs in control conditions, listed in Table 6.

**TABLE 1:** The values of the contractile element parameters for hiPSC-CM-CE (Forouzandehmehr et al., 2021) and hiMCE. F1 and F2 represent DRX:SRX/(DRX:SRX)_control_ ratio in the XB cycling. K_on_, K_np_ and K_pn_ are rate constants for Ca^2+^ binding to troponin, the forward and backward transition rates between Pxb and Nxb states, respectively (Rice et al., 2008). N_perm_ and perm_50_ denote the Hill coefficient and the half-activation constant, respectively, describing the nonlinearity of the cooperativity in Ca^2+^ activation of XBs (Rice et al., 2008). K_offL_ and K_offH_ represent the rate constants affecting Ca^2+^ unbinding from low and high affinity sites on troponin, respectively (Rice et al., 2008). M denotes the mass term in the model of sarcomere by Rice et al. (Rice et al., 2008). Kxb represents the tension scaler detailed in (Forouzandehmehr et al., 2021). Xbmodsp is a species-dpendent XB cycling rate scaler (Rice et al., 2008). H_f_ denotes the rate constant in the forward transition between XB_preR_ and XB_pstR_ (Rice et al., 2008).

#	Parameter	hiPSC-CM-CE	hiMCE (control mode)
1	F1	1	1
2	F2	1	1
3	K_on_ (s^−1^ mM^−1^)	62.5×10^3^	62.5×10^3^
4	K_offL_ (s^−1^)	200	200
5	K_offH_ (s^−1^)	25	25
6	perm_50_	0.6	0.6
7	n_perm_	11.28	11.55
8	K_np_ (s^−1^)	550	550
9	K_pn_ (s^−1^)	50	50
10	K_offmod_	0.5	0.5
11	m (s^2^ µm^−1^)	2 × 10^–5^	2 × 10^–5^
12	kxb	12	13.1
14	xbmodsp	0.2	0.2
15	h_f_ (s^−1^)	2000	2000

This calibrated control/healthy model variant was then modified to describe an HCM mutation and the effect of pharmaceutical mechanical modulators, and in sections 2.2 and 2.3, we explain the tuning of the relevant CE parameters for each scenario. To enlighten the role of these parameters, we describe the XB kinetics (Tran et al., 2010) as follows in relation to Figure 1:

$$\frac{d}{dt}P_{XB}=k_{npt}\times N_{XB}+ap3\times {XB}_{pstR}+am1\times {XB}_{preR}-\left(k_{pnt}+ap1+am3\right)\times P_{XB}$$ (1)

$$\frac{d}{dt}{XB}_{preR}=ap1\times F1\times P_{XB}-am1\times F2\times {XB}_{preR}-ap2\times {XB}_{preR}+am2\times {XB}_{pstR}$$ (2)

$$\frac{d}{dt}{XB}_{pstR}=am3\times P_{XB}+ap2\times {XB}_{preR}-\left(am2+ap3\right)\times {XB}_{pstR}$$ (3)

$$N_{XB}=1-\left(P_{XB}+{XB}_{preR}+{XB}_{pstR}\right)$$ (4)

$where\;k_{npt}$ and $k_{pnt}$ are transition rates between $N_{XB}$ and $P_{XB}$ and responsible for XB cycling activation (Rice et al., 2008). am1 takes into account the Pi-dependent transition rate in the XB and has been defined in (Tran et al., 2010). ap1 is equal to the attachment rate to ${XB}_{preR}$ (Tran et al., 2010) which is given as:

$$f_{ap}\times xbmodsp\times {Qf}_{ap}^{\left(\left(Tmpc-37\right)/10\right)}$$ (5)

$f_{ap}$ value is set to 500 s^−1^ and ${Qf}_{ap}$ is the temperature-dependence set to 6.25 (Rice et al., 2008), in this work. xbmodsp (species-dependent XB cycling rate scaler (Rice et al., 2008)) value is given in Table 1. am3 denotes a thermodynamically constrained transition rate from $P_{XB}$ to ${XB}_{pstR}$ state accounting for MgATP release when the myosin heads transit from weakly to strongly attached XB states detailed in (Tran et al., 2010). ap3 is the MgADP and MgATP-sensitive transition rate from ${XB}_{pstR}$ to $P_{XB}$ (Tran et al., 2010) and is calculated as:

$$ap3=\left[MgATP\right]\times g_{xbt}^{\prime }\times \left(\frac{k_{dADP}+{MgADP}^{*}}{k_{dADP}+\left[MgADP\right]}\right)$$ (6)

Where ${MgADP}^{*}$ denotes the reference (physiological) concentration of MgADP of 36 µM and $k_{dADP}$ is the MgADP dissociation constant detailed in (Tran et al., 2010). $g_{xbt}^{\prime }$ denotes a first order rate constant tuned by Tran et al. (2010) to maintain the validity of original Rice CE model (Rice et al., 2008) under physiological metabolic conditions regarding MgADP kinetics detailed in. am2 is equal to the transition from ${XB}_{pstR}$ to ${XB}_{preR}$ which is proton and MgADP-sensitive (Tran et al., 2010):

$$am2=h_{bt}^{\prime }\times \left[H^{+}\right]\times \left(\frac{k_{dADP}+{MgADP}^{*}}{{MgADP}^{*}}\times \frac{\left[MgATP\right]}{k_{dADP}+\left[MgADP\right]}\right)$$ (7)

Again, $h_{bt}^{\prime }$ represents the adjustment of the rate constant affecting the transition between ${XB}_{preR}$ and ${XB}_{pstR}$ states to include physiological proton and MgADP dependent effects further detailed in (Tran et al., 2010). Following the method proposed in (Margara et al., 2021), we defined F1 and F2 (Eq. (2)) as modulators of the transition between permissive binding state on actin (P_XB_) and strongly bound XBs before isomerized rotation (XB_preR_). In our model, F1 and F2 represent the (DRX:SRX)/(DRX:SRX)_control_ ratio (=1 in control mode) and indirectly affect the ATPase dynamics (Figure 1). ap2 is equal to the forward transition rate between XB_preR_ and XB_pstR_, which is defined as (Rice et al., 2008):

$$h_{f}\times h_{f}md\times xbmodsp\times Q_{hf}^{\left(\left(Tmpc-37\right)/10\right)}$$ (8)

$$h_{f}md=e^{\left(-sign\left({xXB}_{preR}\right)\times hfmdc\times {\left(\frac{{xXB}_{preR}}{x_{0}}\right)}^{2}\right)}$$ (9)

The value for $h_{f}$ is given in Table 1. $Q_{hf}$ represents the temperature dependence, here set to 6.25. $h_{f}md$ incorporates strain dependence into the forward transition rate, $h_{fT}$ . hfmdc is set to five and specifies the extent to which the isomerization rate is influenced by the mean strain of XB_preR_ ( ${xXB}_{preR}$ ). $x_{0}$ is the mean strain (distortion) of XB_pstR_ state when the net motion between actin and myosin filaments is absent. Here, $x_{0}$ is set to 0.007 µm.

Furthermore, ap1 to ap3 and am1 to am3 also affect the time rate of change in the mean strains, Eqs (10), (11), and the steady-state population of strongly bound XB states Eqs (12)–(14) (Tran et al., 2010):

$$\frac{d}{dt}{xXB}_{preR}=0.5\times \frac{dSL}{dt}+\frac{\varphi }{{XB}_{preR}^{DFract}}\left[-\left(ap1\times {xXB}_{preR}\right)+am2\times \left({xXB}_{pstR}-x_{0}-{xXB}_{preR}\right)\right]$$ (10)

$$\frac{d}{dt}{xXB}_{pstR}=0.5\times \frac{dSL}{dt}+\frac{\varphi }{{XB}_{pstR}^{DFract}}\left[ap2\times \left({xXB}_{preR}+x_{0}-{xXB}_{pstR}\right)\right]$$ (11)

$${XB}_{preR}^{DFract}=\frac{am3\times am2+ap3\times ap1+am2\times ap1}{\sum {XB}^{DFract}}$$ (12)

$${XB}_{pstR}^{DFract}=\frac{ap1\times ap2+am3\times ap1+am3\times ap2}{\sum {XB}^{DFract}}$$ (13)

$$\sum {XB}^{DFract}=ap1\times ap2+am3\times am1+am3\times ap2+am3\times am2+ap3\times ap1+am2\times ap1+ap2\times ap3+am3\times am1+ap3\times am1$$ (14)

Where $\frac{dSL}{dt}$ denotes the sarcomere length velocity and φ represents an empirical scaler equal to 2 (Rice et al., 2008). For the detailed explanation of the metabolite-sensitive XB cycling we refer the readers to (Tran et al., 2010).

### The contractile element calibration for HCM model variant

Our baseline CE inherits the main effects of contractile metabolic products such as MgATP, MgADP, inorganic Phosphate (Pi), and H^+^ on the tension development mechanism from the original Tran et al. model (Tran et al., 2010; Tran et al., 2017). We used this baseline model to develop an HCM mutant variant (R403Q) model, with altered myofilament kinetics. This model variant was created by modifying specific metabolic parameters to achieve a simulated state consistent with experimental reports of R403Q HCM (Table 2).

**TABLE 2:** The parameter used in the HCM model variant. Pi_ref denotes the reference value for inorganic phosphate (Pi) in the simulations. Ap2 is a variable influencing detachment of crossbridges. F1 and F2 are coefficients affecting pre-rotational states in XB cycling as also used in (Margara et al., 2021).

#	Parameter	Control value	Value in HCM model variant
1	Pi_ref (mM)	2	18.9
2	MgADP (mM)	36 × 10^–3^	72 × 10^–3^
3	ap2 coef	1	0.315
4	F1	1	1.3
5	F2	1	1.3

To obtain the HCM model variant, we changed F1 and F2 values following (Margara et al., 2021) and in line with the sensitivity test given in Supplementary Figure S4. We also increased the value of MgADP concentration and the reference value of Pi. Finally, we changed the ap2 coefficient regarding the model sensitivity given in Supplementary Figure S2.

### The contractile element calibration for drug-induced effects

Previously, (Margara et al. (2021) assumed in their computational study that MAVA mainly influences the transitions between XB_preR_ and P_XB_ states following the DRX:SRX disturbing theory reported in (Toepfer et al., 2020). This was implemented by introducing F1 and F2 (with default values of 1) coefficients (Eq. (2)) representing DRX:SRX ratios to the time-dependent description of XB_preR_ state.

In addition, we simulated the effect of MAVA not only by altering the values of F1 and F2 but also modifying the parameters listed in Table 1 to obtain a comprehensive and accurate simulation of the effect of 0.5 µM MAVA on our HCM R403Q model variant as given in Table 3. These further modifications, done as a manual parameter tuning, are based on the reported effect of MAVA on Ca^2+^ activation and binding process (Awinda et al., 2020), Pi (Alsulami and Marston, 2020), and ATPase activity (Green et al., 2016). Specifically, changes in K_on_ and n_perm_ values have been made regarding the sensitivity analyses given in (Forouzandehmehr et al., 2021). The F1 and F2 values were changed according to model sensitivity behavior given in Supplementary Figure S4. Further, ap1 and ap3 coefficients and A-E values were obtained by trial and error.

**TABLE 3:** Modifications to the model parameters to simulate the effect of Mavacamten. A, B, C, D, and E are coefficients in Eqs 15–19 affecting P_XB_-XB_PreR_ regulation, Pi-dependent transition in P_XB_-XB_PreR_, XB_preR_-XB_postR_ regulation, Proton-dependent transition in XB_postR_-XB_preR_, and MgATP-dependent transition from XB_postR_-P_XB_, respectively. BL is the baseline value given in Table 1. Default values of A-E, ap1 and ap3 coefs., F1 and F2 are equal to 1.

#	Parameter	Values in 0.5 µM MAVA
1	K_on_ (mM^−1^ s^−1^)	BL × 1.048
2	n_perm_	BL × 0.688
3	A	0.26
4	B	0.4
5	C	5.4
6	D	0.4
7	E	2.39
8	ap1 coef	1.45
9	ap3 coef	0.28
10	F1	0.1
11	F2	0.1

To indicate, A modulates P_XB_ to XB_preR_ transition, B influences Pi-dependent XB_preR_ to P_XB_ transition, C takes effect on XB_preR_ to XB_pstR_ transition, D affects proton-dependent XB_pstR_ to XB_preR_ transition, and E controls MgATP-dependent transition from XB_pstR_ to P_XB_ states. Correspondingly, the proposed modulations (A to E in Table 3) to Eqs (15)–(19), are in line with a disturbed interfilament signaling that affects the force-producing states of XB suggested in the etiology of R403Q (Nag et al., 2015). Our motivation for the A-E coefficient modifications was the role of xbmodsp parameter in contraction relaxation time observed in the sensitivity study before (Forouzandehmehr et al., 2021). As changing xbmodsp solely could not lead to an accurate simulation of impaired relaxation restored by 0.5 µM MAVA, we used A-E values to optimize the distribution of xbmodsp effect on the XB cycling.

$$f_{apt}=f_{ap}\times A\times xbmodsp\times {Qf}_{ap}^{\left(\left(Tmpc-37\right)/10\right)}$$ (15)

$$g_{apt}=g_{ap}\times g_{apslmd}\times B\times xbmodsp\times {Qg}_{ap}^{\left(\left(Tmpc-37\right)/10\right)}$$ (16)

$${h_{ft}=h}_{f}\times h_{f}md\times C\times xbmodsp\times Q_{hf}^{\left(\left(Tmpc-37\right)/10\right)}$$ (17)

$${h_{bt}=h}_{b}\times D\times xbmodsp\times Q_{hb}^{\left(\left(Tmpc-37\right)/10\right)}$$ (18)

$${g_{xbt}=g}_{xb}\times \max\left(g_{xbmd},1\right)\times E\times xbmodsp\times Q_{gxb}^{\left(\left(Tmpc-37\right)/10\right)}$$ (19)

Values of thin filament regulation and XB cycling parameters $h_{b}$ , $g_{ap}$ , $g_{apslmd}$ and temperature dependences $Q_{hb}$ , $Q_{gxb}$ *,* ${Qg}_{ap}$ were directly taken from (Rice et al., 2008). Also, $g_{xbmd}$ is a strain-dependent rate modifier defined in (Rice et al., 2008).

The values of parameters changed in the model to simulate the effects of 5 µM BLEB and 1 µM OM are given in Table 4. F1 and F2 values were obtained with attention to sensitivity plots given in Supplementary Figure S4. Similarly, ap2 and am2 coefficients were found regarding the model behavior shown in Supplementary Figures S2, S3, and S5. Lastly, values of parameters listed in Table 4 rows 7 to 15 were obtained manually according to the sensitivity reports given in (Forouzandehmehr et al., 2021).

**TABLE 4:** The modifications to the parameters to simulate the effects of BLEB and OM. BL: Baseline values of hiMCE model given in Table 1.

#	Parameter	Modifications for 5 µM BLEB	Modifications for 1 µM OM
1	F1	5.015	4.1
2	F2	0.1	0.1
3	Tropreg coef	0.2	0.2
4	ap2 coef	0.012	0.02
5	ap3 coef	0.03	0.03
6	am2 coef	0.25	0.15
7	K_on_ (mM^−1^ s^−1^)	-	BL × 1.28
8	n_perm_	-	BL × 1.182
9	perm_50_	BL × 1.33	BL × 1.33
10	K_pn_ (s^−1^)	-	BL × 0.2
11	K_np_ (s^−1^)	-	BL × 1.182
12	K_offmod_	-	BL × 0.52
13	K_offL_ (s^−1^)	-	BL × 1.75
14	K_offH_ (s^−1^)	-	BL × 0.6
15	h_f_ (s^−1^)	-	BL × 2

Moreover, to capture the dose-dependent effect of BLEB and OM on the normalized tension, based on model sensitivity tests (Supplementary Figures S2-S5) and previous sensitivity analyses (Forouzandehmehr et al., 2021), we identified the main variable of the CE governing the maximum developed tension, ap2 , and identified coefficient values accordingly (Supplementary Tables S1 and S2). Supplementary Figures S2-S5 show the sensitivity of tension-Ca^2+^ relationships, active tensions, and ATPase rates to ap2 , am2 , and R (=F1 = F2) coefficients. Our HCM and drug-induced calibrations are unique sets obtained from an informed manual tuning, with e.g., 10%, 20%, 50% increments, started with a first guess within assumed boundaries (e.g., 1/100 to 5.4 times of baseline values). The examples of the results showing the increments and the model response are given in Supplementary Figures S2-S5. Overall, these sensitivity analyses combined with our previous sensitivity investigations on the CE of our previous model (Forouzandehmehr et al., 2021) were the basis of the informed manual parameter tunings done to obtain HCM- and drug-induced calibrations in this work.

### The experimental data for calibrations and validations

Table 5 gives the experimental data using which the results of this work have been calibrated and validated.

**TABLE 5:** The experimental data used for calibration of the model and validation of the simulated results.

Type	Experiment	Cell/tissue type	Observation	Figure/Table	Preparation data
Calibration	Toepfer et al. (2020)	hiPSC-CMs	33% increase of tension relaxation in R403Q	Figure 3C	hiPSC-CM cell lines at day 30 post-differentiation
			Corrected tension relaxation due to MAVA in R403Q		
			The fractional cell shortening in R403Q	Figure 3F	
	(Green et al. (2016); Toepfer et al. (2020))	hiPSC-CMs, Murine	Reduction in fractional cell shortening due to MAVA	Figure 3F	hiPSC-CM cell lines at day 30 post-differentiation, isolated adult rat ventricular cardiomyocytes treated with increasing concentrations of MAVA
	(Green et al. (2016); Awinda et al. (2020))	Murine, Human	Reduction in maximum tension due to MAVA	Figure 3C	Isolated adult rat ventricular cardiomyocytes, skinned human myocardial strips
	Kampourakis et al. (2018)	Rat	Change in pCa50, Hill coefficient, and maximum tension due to OM and BLEB	Table 7 and Figure 4A	Demembranated rat ventricular trabeculae
			OM and BLEB dose-dependent contractile response	Figures 4D,E	
Validation	Sewanan et al. (2019)	hiPSC-CMs	The unaffected CaT in R403Q	Figure 3B	Engineered heart tissues made of hiPSC-CMs on decellularized porcine left ventricular tissue blocks
	(Nag et al. (2015); Sarkar et al. (2020))	Human, hiPSC-CMs	Negligible change in ATPase in R403Q	Figure 3D	Human *β*-cardiac myosin
	Green et al. (2016)	Murine	Unchanged CaT and pCa_50_ in Tension-pCa curve due to MAVA	Figure 3B, Fig. S1	Isolated adult rat ventricular cardiomyocytes
	Rohde et al. (2018)	Human, Porcine, Bovine, and Murine	Reduction in ATPase rate due to MAVA	Figure 3D	Human *β*-cardiac myosin, Porcine and Bovine ventricular myosin, mouse cardiac myofibrils
	Kawas et al. (2017)				
	Green et al. (2016)				
	Gollapudi et al. (2021)				
	Kawas et al. (2017)	Human & Bovine	Slowed relaxation in ATPase rate due to MAVA	Figure 3D	Human and bovine cardiac myosins
	Szentandrassy et al. (2016)	Canine	Change in APD due to 1 µM OM	Figure 5A	Left ventricular single canine myocytes
	(Bakkehaug et al. (2015); Utter et al. (2015))	Murine Porcine	ATPase basal value increase due to OM	Figure 5B	*Ex vivo* mouse and *in vivo* pig hearts

## Results

### The metabolite-sensitive model of hiPSC-CMs

All the equations were solved using ode15 s integrator of MATLAB with a maximum step size of 10^–3^ and an initial step size of 2 × 10^–5^. To reach the steady state, the results were obtained after 800 beats paced at 1 Hz unless otherwise mentioned.

**FIGURE 2:**
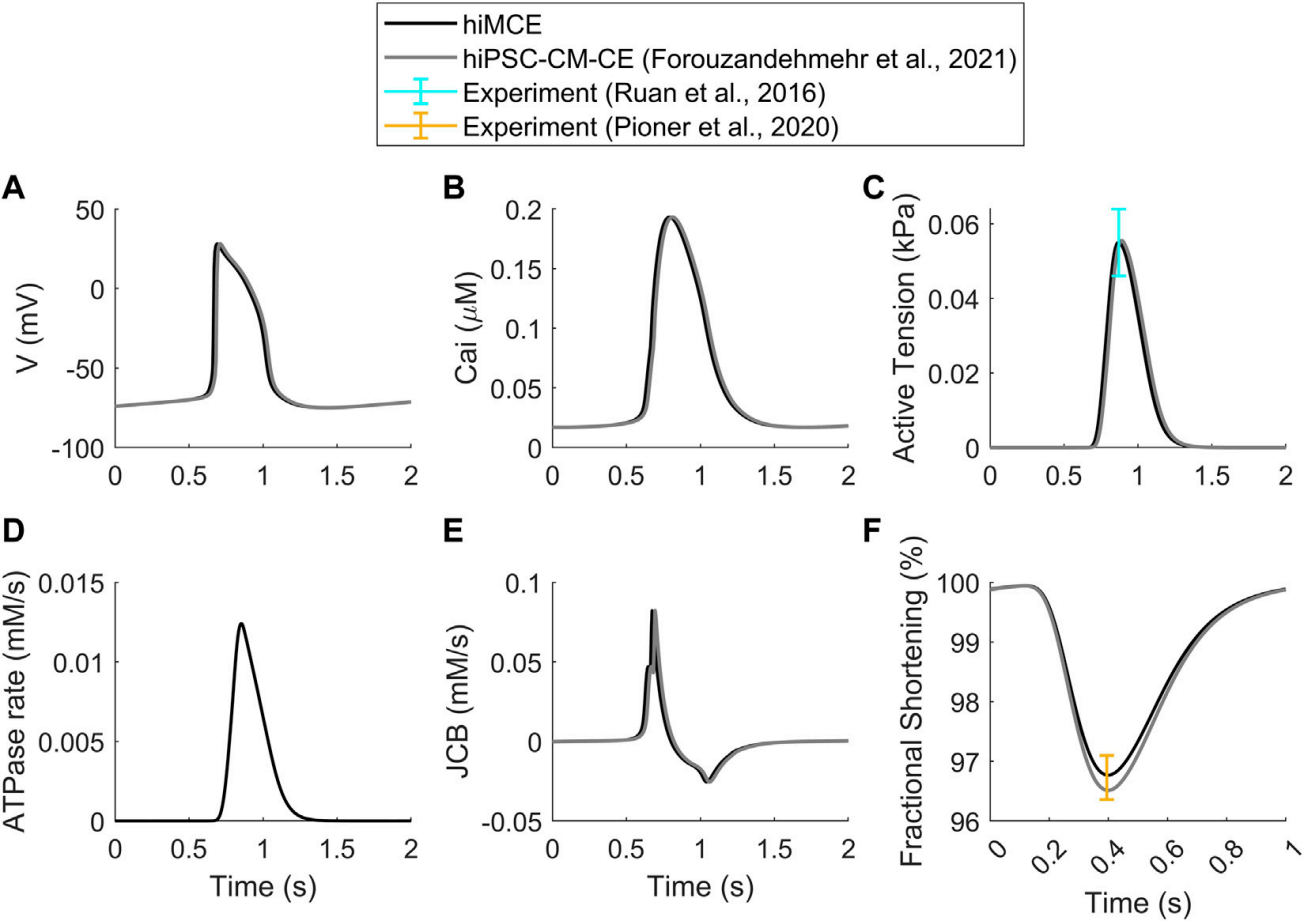
Standard results of the model in spontaneous beating: Action Potentials **(A)**, Ca^2+^ Transients **(B)**, Active Tensions **(C)**, ATPase rate **(D)**, Flux of Ca^2+^ towards the contractile element **(E)**, Fractional cell shortening at 1 Hz pacing **(F)**. Cited works: (Ruan et al., 2016; Pioner et al., 2020; Forouzandehmehr et al., 2021).

First, we show that our computational model can correctly simulate the main AP, Ca^2+^ transients (CaTs), and active tension (AT) biomarkers as had been simulated by our previous electromechanical hiPSC-CM-CE model (Forouzandehmehr et al., 2021). As Table 6 shows, our new metabolite-sensitive hiMCE model is able to simulate the main biomarkers within the experimental ranges in the validation datasets. The increased thermodynamic detail of the CE did not significantly alter the biomarker values compared to our previous reparameterization (Forouzandehmehr et al., 2021) and the original Paci et al. hiPSC-CM model (Paci et al., 2020). Also, Figure 2 shows the contractility characteristics simulated using the hiMCE model are consistent with the previously validated results, while also illustrating selected fundamental outputs (Ruan et al., 2016; Pioner et al., 2020).

**TABLE 6:** Action potential (AP), Ca^2+^ transients (CaT), and active tension (AT) calculated biomarkers in spontaneous condition and their comparison with Paci2020 and hiPSC-CM-CE model (i.e. no metabolite-sensitive CE) and the experimental values (Paci et al., 2018; Paci et al., 2020). APA: AP amplitude, MDP: maximum diastolic potential, CL: cycle length, dV/dt max: maximum upstroke velocity, APD_10_ and APD_30_ and APD_90_: AP duration at 10, 30, 90% of repolarization, respectively, AP Tri: AP triangulation index. The simulated biomarkers of CaT are DURATION: duration of the transient, tRise_10, peak_: time to peak, tRise_10, 50_ and tRise_10, 90_: rise time from 10 to 50% and 90% of maximum threshold, respectively, and tDecay_90,10_: decay time from 90 to 10%. AT: Active tension, RT_50_: time from peak contraction to 50% of relaxation, %FS: percent of fractional shortening. The experimental ranges for contraction biomarkers are from (Forouzandehmehr et al., 2021). The third column is taken directly from the original Paci2020 publication (Paci et al., 2020).

No.	Biomarker	Paci2020	hiPSC-CM-CE	hiMCE	Exp. Value (Mean ± SD)
1	APA (mV)	102	103	103	104 ± 6
2	MDP (mV)	-74.9	-75.0	-75.0	-75.6 ± 6.6
3	AP CL (ms)	1712	1644	1644	1700 ± 548
4	dV/dt max (V/s)	20.5	23.9	24.0	27.8 ± 26.3
5	APD10 (ms)	87.0	95.0	95.1	74.1 ± 26.3
6	APD30 (ms)	224	238	238	180 ± 59
7	APD90 (ms)	390	403	403	415 ± 119
8	AP Tri	2.8	2.9	3	2.5 ± 1.1
9	CaT DURATION (ms)	691	693	693	805 ± 188
10	CaT tRise10, peak (ms)	184	163	163	270 ± 108
11	CaT tRise10,50 (ms)	54.9	46.2	45.9	82.9 ± 50.5
12	CaT tRise10,90 (ms)	118	102	102	167 ± 70
13	CaT tDecay90,10 (ms)	341	343	343	410 ± 100
14	AT magnitude (kPa)	-	0.055	0.055	0.055 ± 0.009
15	RT50 (ms)	-	161	158	158 ± 12.1
16	%FS	-	3.45	3.23	3.27 ± 0.37

### Hypercontractility in R403Q HCM and mavacamten

**FIGURE 3:**
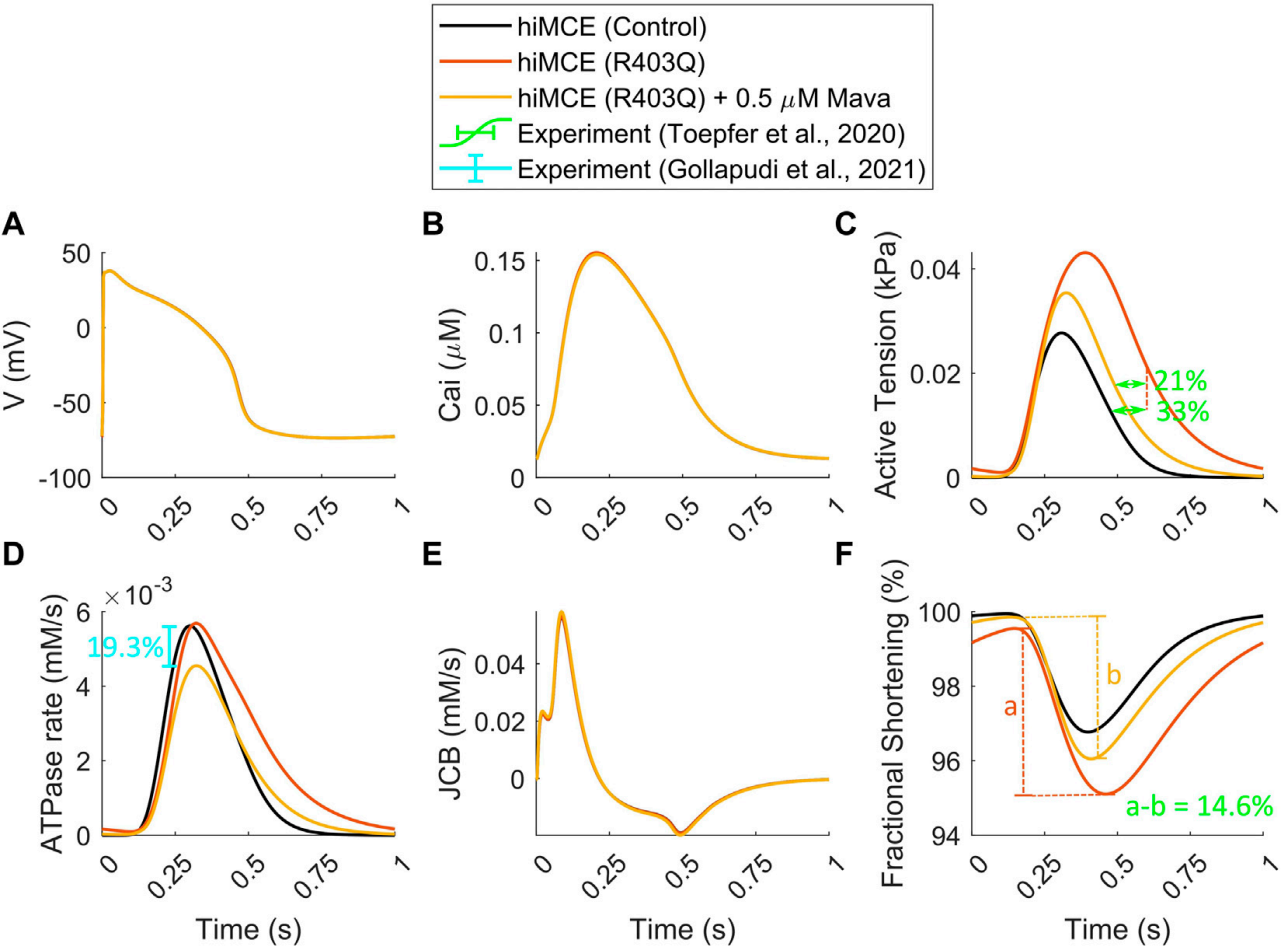
Simulated action potential **(A)**, Calcium transients **(B)**, active tensions **(C)**, ATPase rate **(D)**, Flux of Ca^2+^ towards the myofilament **(E)**, and fractional cell shortenings **(F)** in R403Q hypertrophic cardiomyopathy and Mavacamten modes (All simulations were done at 1 Hz pacing). The percents of prolonged tension relaxation in R403Q mode **(C)**, the reduction in tension relaxation due to MAVA **(C)**, the reduction in fractional shortening in R403Q mode due to MAVA **(F)**, and the reduction in ATPase rate due to MAVA **(D)** agree with the experimental data (Toepfer et al., 2020; Gollapudi et al., 2021).

In order to simulate the abnormal prolonged relaxation in the developed active tension due to R403Q HCM mutation, Margara et al. (2021) hypothesized a feedback from XB cycling to the thin filament activation. To investigate further, using the parameter values in Table 2 and consistent with the metabolic data detailed in section 2.2, we simulated the active tension and ATPase rate in R403Q HCM model variant. The CaT morphology remains unchanged in the HCM R403Q mode (Figure 3B), consistently with experimental data reported for hiPSC-CMs in (Sewanan et al., 2019). Interestingly, the results in Figure 3C suggest that including energetics in the CE reacts to the pathological changes due to HCM and can correctly predict the prolonged relaxation in the developed active tension (∼33%), consistently with *in vitro* hiPSC-CMs data (Toepfer et al., 2020). Moreover, the increased fractional cell shortening (∼40%) due to the R403Q mutation is consistent with experimental measurements in (Toepfer et al., 2020). Of note, the model also correctly predicts the negligible change in the ATPase activity (Figure 3D), consistently with the experimental data (Table 5) (Nag et al., 2015; Sarkar et al., 2020).

To simulate the electro-mechano-energetic effect of 0.5 µM MAVA, we used the model calibration values listed in Table 3. We have assumed that MAVA would shift the elevated metabolites in the HCM model variant, Pi and MgADP, towards their baseline values. Our model could accurately predict the unaffected CaTs due to MAVA as reported experimentally earlier (Green et al., 2016). Further, the order of reduction in the simulated ATPase rate (19.3%) due to 0.5 µM MAVA, Figures 3D, 3is within the reduction range, 17.9–28.5%, reported in previous experimental ATPase activity measurements (Gollapudi et al., 2021). Also, the model consistently predicts the reduction in the relaxation phase in the ATPase rate (Figure 3D) (Kawas et al., 2017). Notably, our simulations quantitatively capture the reduction in the fractional cell shortening and prolonged tension relaxation due to R403Q mutated hiPSC-CMs after 0.5 µM MAVA (14.6% and 20.9%, respectively), consistently with recent experimental measurements (Toepfer et al., 2020). Finally as shown in Supplementary Figure S1, the CE model accurately predicts the unchanged pCa_50_ in the tension-Ca^2+^ relationship consistent with the experimental data for 0.5 µM MAVA (Green et al., 2016).

### Simulated effects of omecamtiv mecarbil and blebbistatin

**FIGURE 4:**
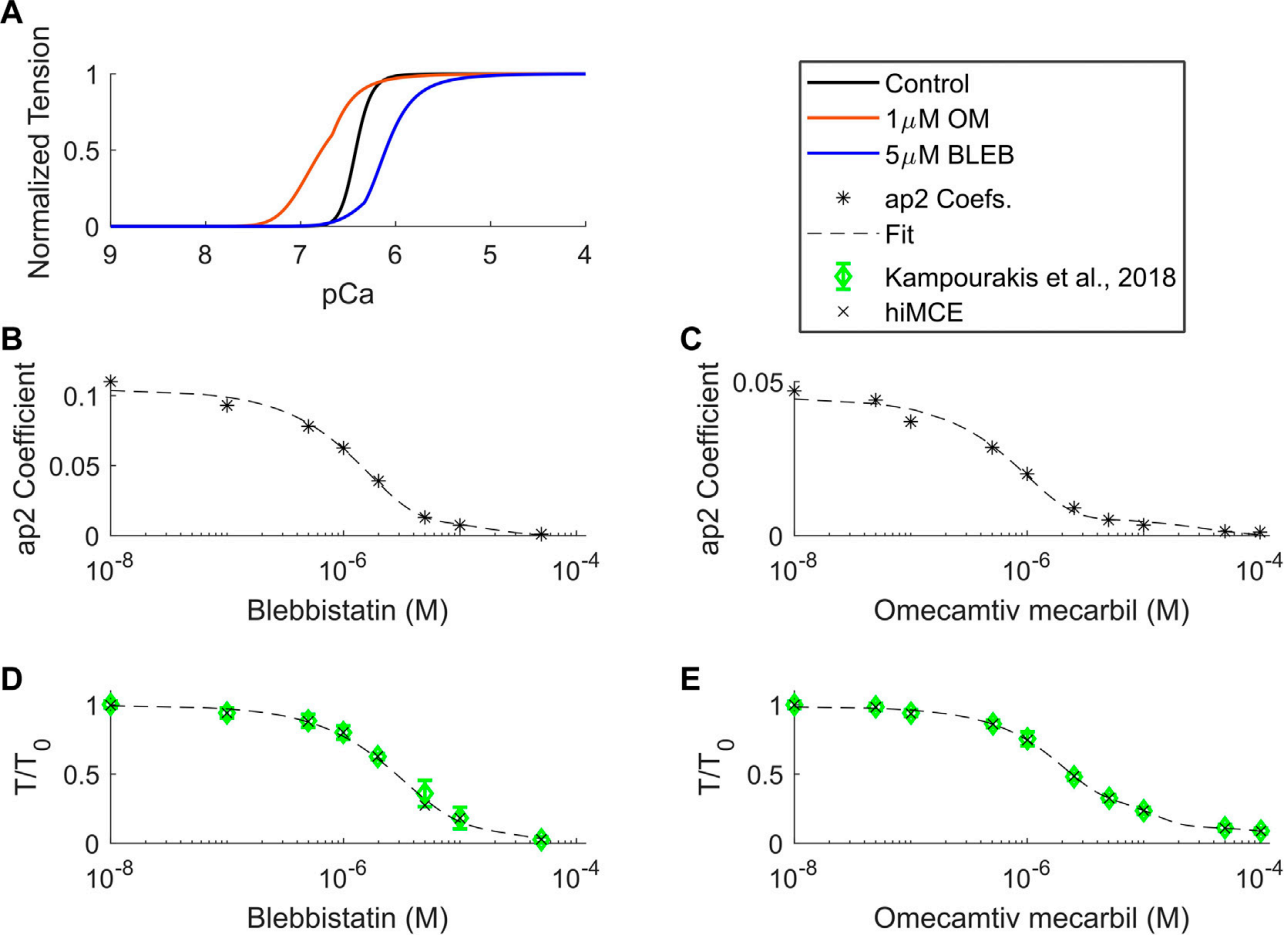
Calcium-tension relationships in control and drug-induced modes in isometric condition **(A)**, ap2 coefficients found for different BLEB and OM concentrations **(B,C)**, and dose-dependent tension-Ca^2+^ relationships of BLEB **(D)** and OM **(E)** in isometric conditions. OM: Omecamtive mecarbil. BLEB: Blebbistatin. Experimental data from (Kampourakis et al., 2018). T_0_: isometric force in the absence of drugs.

Using the CE parameter values listed in Table 4, we simulated the effect of 5 µM BLEB and 1 µM OM. As Table 7 shows, the drug-induced calibration of the hiMCE model results in accurate predictions of Ca^2+^ sensitive effects of 5 µM BLEB and 1 µM OM consistent with experimental data (Kampourakis et al., 2018) as also Figure 4A qualitatively confirms. Additionally, the selected values for the coefficients of the tension governing variables in the CE, ${ap}_{2}s$ (Figures 4B,C and Supplementary Tables S1, S2), leads to the correct dose-dependent prediction for BLEB and OM (Figures 4D,E) and the expected inverse Hill curves reported experimentally (Kampourakis et al., 2018).

**TABLE 7:** Experimental data (Kampourakis et al., 2018) and hiMCE results due to the effect of 1 µM OM and 5 µM BLEB. pCa_50_ represents the -log of the Ca^2+^ concentration associated with 50% of maximum tension. cTnC-E: data from cardiac troponin C (cTnC) E-helix obtained by a rhodamine probe. cRLC-E: Data from a probe connected to the myosin regulatory light chain (RLC).

#	Item	Kampourakis et al., 2018	Hi-MCE model (%)
1	Increase in pCa50 due to 1 µM OM	5.4–6.9% (cRLC-E)	5.8
		3.8–6.4% (cTnC-E)	
2	Decrease in pCa50 due to 5 µM BLEB	2.1–5.5% (cRLC-E)	4.8
		2.6–5.7% (cTnC-E)	
3	Reduction of Hill coef. Due to 1 µM OM	53.2–64.5% (cRLC-E)	58.9
		57.1–68.1% (cTnC-E)	
4	Reduction of Hill coef. Due to 5 µM BLEB	59.5–73.5% (cRLC-E)	49.9
		44.8–58.3% (cTnC-E)	
5	Reduction in max tension due to 1 µM OM	0–29% (cRLC-E)	25.3
		18–47% (cTnC-E)	
6	Reduction in max tension due to 5 µM BLEB	66–90% (cRLC-E)	76.4
		64–80% (cTnC-E)	

**FIGURE 5:**
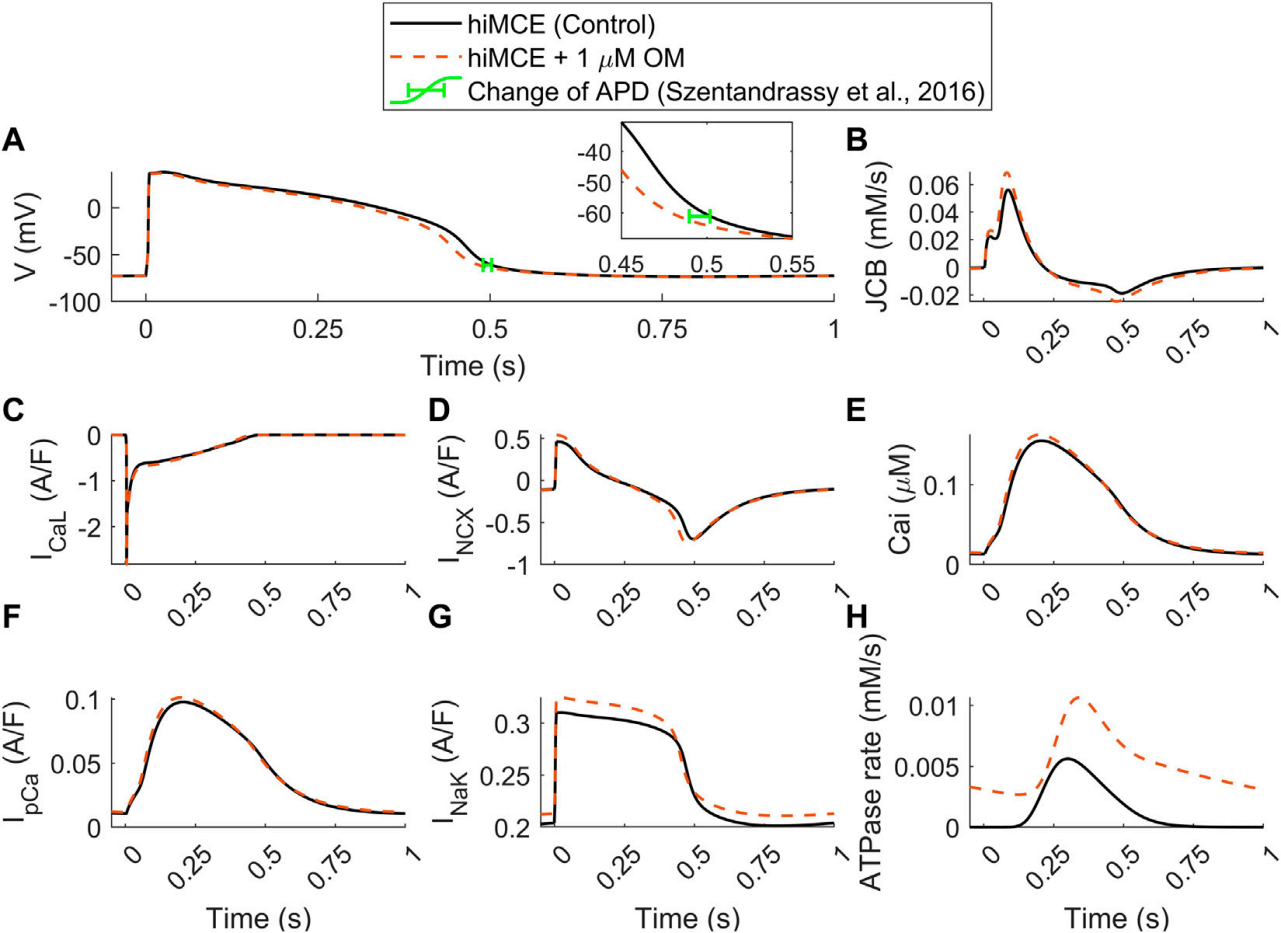
Predicted effect of 1 µM OM by hiMCE on action potentials **(A)**, Ca^2+^ flux towards the myofilament **(B)**, L-type Ca^2+^ current **(C)**, Na^+^/Ca^2+^ exchanger I_NCX_ **(D)** Ca^2+^ transients **(E)**, sarcolemmal Ca^2+^ pump current **(F)**, Na^+^/K^+^ pump **(G)**, and ATPase rates **(H)**. The change of APD has been considered calculating APD_90_ in agreement with experimental data reported in (Szentandrassy et al., 2016). OM: Omecamtiv mecarbil.

Moreover, our model predicts an insignificant reduction in AP duration (3.4%) due to 1 µM OM (Figure 5A), evaluated by calculating APD_90_ values (Figure 5A). This translates to 17 ms reduction in APD_90_ (502–485 ms) which is consistent with the order of APD_90_ reduction due to 1 µM OM, 12.2 ms, reported for canine cardiomyocytes at 1 Hz pacing (Szentandrassy et al., 2016). Also, the simulated increase in the basal ATPase rate due to 1 µM OM (Figure 5H) is qualitatively consistent with the experimental data reported before (Bakkehaug et al., 2015; Utter et al., 2015).

Our model predicts a 23% increase in the amplitude of the Ca^2+^ flux towards the myofilament, JCB (Figure 5B). The subsequent accumulation of intracellular Ca^2+^ is seen as a 4.5% increase in CaT peak (Figure 5E). Interestingly, the steady-state alterations in sarcolemmal Ca2+ transport are very subtle: virtually unchanged I_CaL_ (Figure 5C) and only very slightly increased I_pCa_ (Figure 5F). Whereas there is a more substantial 17% increase in the amplitude of the I_NCX_ (Figure 5D). The enhanced reverse mode of I_NCX_ causes accumulation of intracellular Na^+^ (7.04 vs. 7.43 mM) that promotes a stronger repolarizing I_NaK_ (Figure 5G). This appears to be the mechanism that causes the subtle yet visible 3.4% decrease in the AP duration (Figure 5A), consistently with the reported experiments suggesting OM as a safe compound on cardiac electrophysiology in clinically tolerated doses (Szentandrassy et al., 2016). These predictions can be insightful regarding the consequences of the disturbed interfilament signaling that OM elicits in the XBs.

## Discussion

### HCM and energetics of contraction

Cardiomyocytes, with no self-renewal capacity, must provide two billion beats during an average lifetime for which the cardiac muscle requires a significant amount of energy, 6 kg of ATP per day (Ingwall, 2002). This energy consumption is predominantly due to the function of sarcomeres in contraction. Therefore, the pathological conditions directly caused by sarcomeric mutations, such as R403Q HCM, necessitate studying contractile function of cardiomyocytes regarding cardiac metabolism. Our analysis demonstrates that the incorporated scheme of the metabolite-sensitive CE is able to capture the impaired (prolonged) tension relaxation, ∼33%, due to the R403Q mutation. Interestingly, with the energetics included, the additional feedback from XB cycling to the thin filaments, proposed previously by Margara et al. (2021), was not necessary to replicate the altered relaxation. This further highlights the importance of considering (patho)physiologically constrained metabolite-sensitive computational models in the investigation of sarcomeric cardiomyopathies.

### HCM and drug-induced model calibrations

Contractile energetics become highly important when studying promising drugs reported in HCM clinical trials such as MAVA, BLEB, and OM. As our results show, the quantitatively valid simulation of the effect of MAVA, BLEB, and OM, in single dose or dose-dependently, could not be done without the calibration of parameters in the CE that directly or indirectly affect the energetics (Tables 3, 4, S1, and S2). Markedly, one of the important insights of this study stems from the parameters involved in the calibration of the model for the simulation of 0.5 µM MAVA. We took the MAVA modeling one step further by calibrating the CE altering parameters affecting Pi-dependent transition between permissive binding state on actin (P_XB_) and the strongly bound XBs before isomerized rotation (XB_preR_) state. Also, we modulated Ca^2+^ binding and sensitivity of the CE, and MgATP-dependent transitions between P_XB_ and XBs in strongly bound post isomerized rotation state (XB_postR_) in accord with experimental metabolic reports (Green et al., 2016; Alsulami and Marston, 2020; Awinda et al., 2020). Towards decoding the precise drug mechanism of action, the modulations proposed here to explain the effect of MAVA (Table 3) implies that, alongside altering the disturbed DRX:SRX ratio, MAVA might also induce a new interfilament equilibrium, modulating tension-producing and energetic terms that explicitly affect the reverse transition at play between XB_preR_ and XB_postR_ affecting the strain-dependent isomerization of myosin heads. This possible pharmacological insight emerging from our model is interesting as MAVA mechanism of action inherently shifts the R403Q impaired metabolism towards normal regulation and this involves the impaired proton-dependent transition in R403Q mode (Toepfer et al., 2020).

The drug- and HCM-related calibrations presented in this work are in line with the proposed OM and BLEB structure-function relationships detailed in (Kampourakis et al., 2018). To enumerate, Kampourakis et al. (2018) have implied that OM-bound myosin heads relocate the tropomyosin to its on state when binding to actin in the absence of Ca^2+^ bound to troponin. Importantly, the XB activation, which is due to the effect of OM, has been significantly attributed to the stabilizing of the ON state of thick filaments. Further, these stabilized ON positions in thick filaments have been considered to promote an ON thin filament state through preventing tropomyosin returning to their off positions. This has been translated in our model by modifying k_off_ constants which are the rate constants affecting Ca^2+^ unbinding from low and high affinity sites on troponin. These transition rates affect regulatory sites on cardiac troponin leading to activation of XB cycle (Tran et al., 2010).

On the other hand, at intermediate Ca^2+^ in the physiological range (pCa nine to 4.3), OM activates actins along with the thick filaments. In our model, this has been translated by modifying K_on_, K_np_ and K_pn_ rate constants for Ca^2+^ binding to troponin, forward and backward transition rates between P_xb_ and N_xb_ states, respectively (Table 4). Further, the constant rates directly affecting Ca^2+^ bound troponin thin filaments regulation induced by Ca^2+^ bound troponin (K_on_, n_perm_, k_offL_, k_offH,_ k_pn_, k_np_, and k_offmode_) have only been calibrated towards activation for OM and they are missing in BLEB calibration as BLEB does not switch both filaments to their ON states (Kampourakis et al., 2018). Further, modification of coefficient of Tropreg variable, the fraction of actins with Ca^2+^ bound, detailed in (Rice et al., 2008), in both OM and BLEB calibrations is consistent with the reported effect of these drugs as both OM and BLEB generally greatly decrease the Ca^2+^ activation co-operativity (Kampourakis et al., 2018).

In the HCM variant model, the significant increase in Pi concentration is consistent with the experimental metabolic reports of HCM R403Q and R92Q mutations in animal mouse models (Spindler et al., 1998; Abraham et al., 2013). Moreover, the impaired coronary perfusion due to HCM has been related to an abnormal energy reproduction that contributes to elevation of ADP and Pi (Sequeira et al., 2019), which is also consistent with conservation of phosphate and creatin reaction (Tran et al., 2010). Congruently, the HCM mutation-induced alterations in myofilament kinetics lead to increase in ADP-mediated products (van der Velden et al., 2018). Therefore, we increased the MgADP concentration within physiological ranges (Tran et al., 2010). Notably, the Ca^2+^ dependence in the activation of myofilaments for HCM and dilated cardiomyopathy has been shown to be altered in a similar fashion due to OM and BLEB influence (Spudich, 2014), implying that the etiology of HCM includes a disturbed actomyosin signaling. In addition, the underlying mechanism of HCM-induced hypercontractility, including R403Q HCM, has been explained in light of thick filament structural alterations and the tension generation (Alamo et al., 2017; Nag et al., 2017; Trivedi et al., 2018). Granted that, since with change in ap2 coefficient (Table 2) the XB cycling machinery could closely capture the HCM behavior (Figure 3), our model potentially attributes the HCM-induced disturbed interfilament signaling to a distortion-dependent forward transition from ${XB}_{preR}$ to ${XB}_{pstR}$ state and the subsequent effect due to population of strongly bound XB states in the steady-state condition. This could imply that the HCM-induced disturbed actomyosin signaling might stem from a misregulated isomerization of myosin heads from pre-rotated to post-rotated force generating state affecting the strain induced in the myosin neck region. Analogically, the same process might explain how OM, BLEB, and Mava contribute to a new interfilament balance, restoring normal XB cycling tension generation. This hypothesis based on our model predictions could be insightful for accelerating the future drug development for sarcomere cardiomyopathies and mutation-specific HCM.

We have introduced a mechanistic solution to incorporate the dose-dependent effect of Blebbistatin and Omecamtiv mecarbil (Figure 4) consistent with experiments (Kampourakis et al., 2018) focusing on ap2 . Explicitly, the inverse Hill function trend observed in dose-dependent effects of OM and BLEB (Kampourakis et al., 2018) also reflects in the values of ap2 coefficients obtained for the studied concentrations (Figures 4B–E). Moreover, changes in F2, hf, and ap2 (Table 4) suggest that OM also favors the rapid detachment of XBs as another contributor to the disturbed actomyosin coupling involved in OM mechanism of action. Furthermore, the accurate simulation of shortened APD due to 1 µM OM combined with elevated basal ATPase rate values further signifies that inclusion of metabolite-sensitive transitions in the XB cycling of the CE cannot be ignored in precision medicine, especially when simulating the effects of drugs whose main mechanism of action impacts ATPases.

### Limitations and future works

The developed mathematical model naturally has some limitations and potentials for advancements in the future studies. Firstly, as we use ODEs instead of computationally expensive PDEs, the cooperative spatial interactions between regulatory proteins and XB action have been approximated with a mean-field technique (Rice et al., 2008). Secondly, we assumed that 0.5 µM MAVA restores the elevated MgADP and Pi in HCM model variant to their basal values (listed in Table 2). Although this assumption is consistent with MAVA mechanism of action (Green et al., 2016), it is still a simplification. Thirdly, one fundamental limitation of hiPSC-CMs is that they rely on glycolytic metabolism, in contrast to the fatty acid-based metabolism of native human adult ventricular cardiomyocytes. A further source of energetic dissimilarities is the differences between surrounding medium *in vitro* vs. *in vivo* conditions. Given these and as our model does not include energy production process, we consider the metabolic differences out of the scope of this work. As detailed *in vitro* data on hiPSC-CM metabolism emerges, our modeling efforts serve as a solid basis for the next phase of cardiomyocyte models with energy production included.

The structural immaturity and special sarcomere alignment and performance in hiPSC-CMs and its effect on the HCM and drug-induced studies are important. The core of our HCM variant and MAVA calibration is based on hiPSC-CMs *in vitro* data obtained at day 30 post-differentiation, indicating cardiomyocytes maturation (Toepfer et al., 2020). In this work (Toepfer et al., 2020), to validate the hiPSC-CMs finding regarding *in vivo* data, the authors conducted parallel analyses on mouse HCM model and human HCM cardiac tissues. The comparison revealed that each HCM variant (HCM mice and hiPSC-CMs) caused hypercontractility with respect to its WT model (Figure 3 C&I in (Toepfer et al., 2020)). Furthermore, the prolonged contractile relaxation also was observed in mouse cardiomyocytes and hiPSC-CMs (Figures 3D,J in (Toepfer et al., 2020)). In addition, the dose-dependent decrease in hypercontractility and myosin population in DRX due to MAVA was observed in mouse cardiomyocytes and hiPSC-CMs (Figures 3E,K in (Toepfer et al., 2020)). All in all, Figure 3 in Toepfer et al. (2020) shows that HCM-induced and drug induced effects on hiPSC-CMs are consistent with the corresponding trend observed in mouse WT and R403Q cardiomyocytes variants. Although, our priority in calibration and validation of our results was using hiPSC-CMs *in vitro* data wherever available, all the abovementioned points imply that validation and calibration of HCM and drug-induced results with cell lines other than hiPSC-CMs are still valid and legitimate approaches.

Furthermore, as our computational model is 0D, it does not explicitly account for the disorganization of sarcomeres typical for hiPSC-CMs. This could be an interesting and valuable future direction for HCM computational studies as more experimental data becomes available.

Finally, the model can benefit from the inclusion of a metabolite-sensitive formulation of the intracellular SERCA pump, as a key ATP-dependent transporter. However, as the focus of this work was sarcomeric cardiomyopathies we have considered it out of scope here.

## Conclusion

As cardiac precision medicine arises (Niederer et al., 2019; Paci et al., 2021; Forouzandehmehr et al., 2022), the demand for comprehensive computational models capable of performing high throughput pharmacological investigations heightens. This works proposes a novel metabolite-sensitive computational model of hiPSC-CMs electromechanics with demonstrated capacity to simulate sarcomeric cardiomyopathies and the compounds directly affecting the myosin dynamics considering the metabolic pathways. The mechanistic method offered for simulating the effects of HCM and drugs in this work lends insights upon the molecular interactions in contractile function and advance our pathophysiological understanding of the development of future therapeutics for HCM.

## Data Availability

The original contributions presented in the study are included in the article/supplementary material, further inquiries can be directed to the corresponding author.
