# Supplementary material for: Altered contractility in mutation-specific hypertrophic cardiomyopathy: A mechano-energetic in silico study with pharmacological insights
Source: Front Physiol. 2022 Oct 31;13:1010786. doi: 10.3389/fphys.2022.1010786 (PMC9659818; doi:10.3389/fphys.2022.1010786)
Supplement: Supplementary file 1 [file Table1.docx]

Supplementary Material

For the control mode of the model, the basis of the calibration was tuning the model parameters within ±25% of the CE baseline values used in [1] as the constraint consistently with the parameters adjustments that have been reported previously [2–5]. For the calibration of the HCM and drug-induced variants of the model, the unique parameter sets were explored with e.g. ±10%, ±20%, and ±50% increments within 1/100 to 5.4 times of baseline values following Zile and Trayanova’s work [5]. The examples of the sensitivity analyses that informed these manual tunings can be found in [1] and in Figs. S2-S5.

# Supplementary Figures and Tables

Table S1 ap2 coefficients at different Blebbistatin (BLEB) concentrations and simulated normalized tensions. T is the isometric force and T_0_ is the isometric force in the absence of drugs.

| # | BLEB Concentration (M) | ap2 Coefficient | Simulated T/T_0_ |
| --- | --- | --- | --- |
| 1 | 1×10^-8^ | 0.1100 | 0.9964 |
| 2 | 1×10^-7^ | 0.0930 | 0.9407 |
| 3 | 5×10^-7^ | 0.0780 | 0.8796 |
| 4 | 1×10^-6^ | 0.0626 | 0.8003 |
| 5 | 2×10^-6^ | 0.0390 | 0.6270 |
| 6 | 5×10^-6^ | 0.0130 | 0.2735 |
| 7 | 1×10^-5^ | 0.0074 | 0.1816 |
| 8 | 5×10^-5^ | 0.0010 | 0.0288 |

Table S2 ap2 coefficients at different Omecamtiv mecaribil (OM) concentrations and simulated normalized tensions. T is the isometric force and T_0_ is the isometric force in the absence of drugs.

| # | OM Concentration (M) | ap2 Coefficient | Simulated T/T_0_ |
| --- | --- | --- | --- |
| 1 | 1×10^-8^ | 0.0470 | 0.9996 |
| 2 | 5×10^-8^ | 0.0440 | 0.9827 |
| 3 | 1×10^-7^ | 0.0370 | 0.9360 |
| 4 | 5×10^-7^ | 0.0286 | 0.8604 |
| 5 | 1×10^-6^ | 0.0200 | 0.7462 |
| 6 | 2.53×10^-6^ | 0.0090 | 0.4848 |
| 7 | 5×10^-6^ | 0.0051 | 0.3260 |
| 8 | 1×10^-5^ | 0.0034 | 0.2366 |
| 9 | 5×10^-5^ | 0.0014 | 0.1088 |
| 10 | 1×10^-4^ | 0.0011 | 0.0884 |


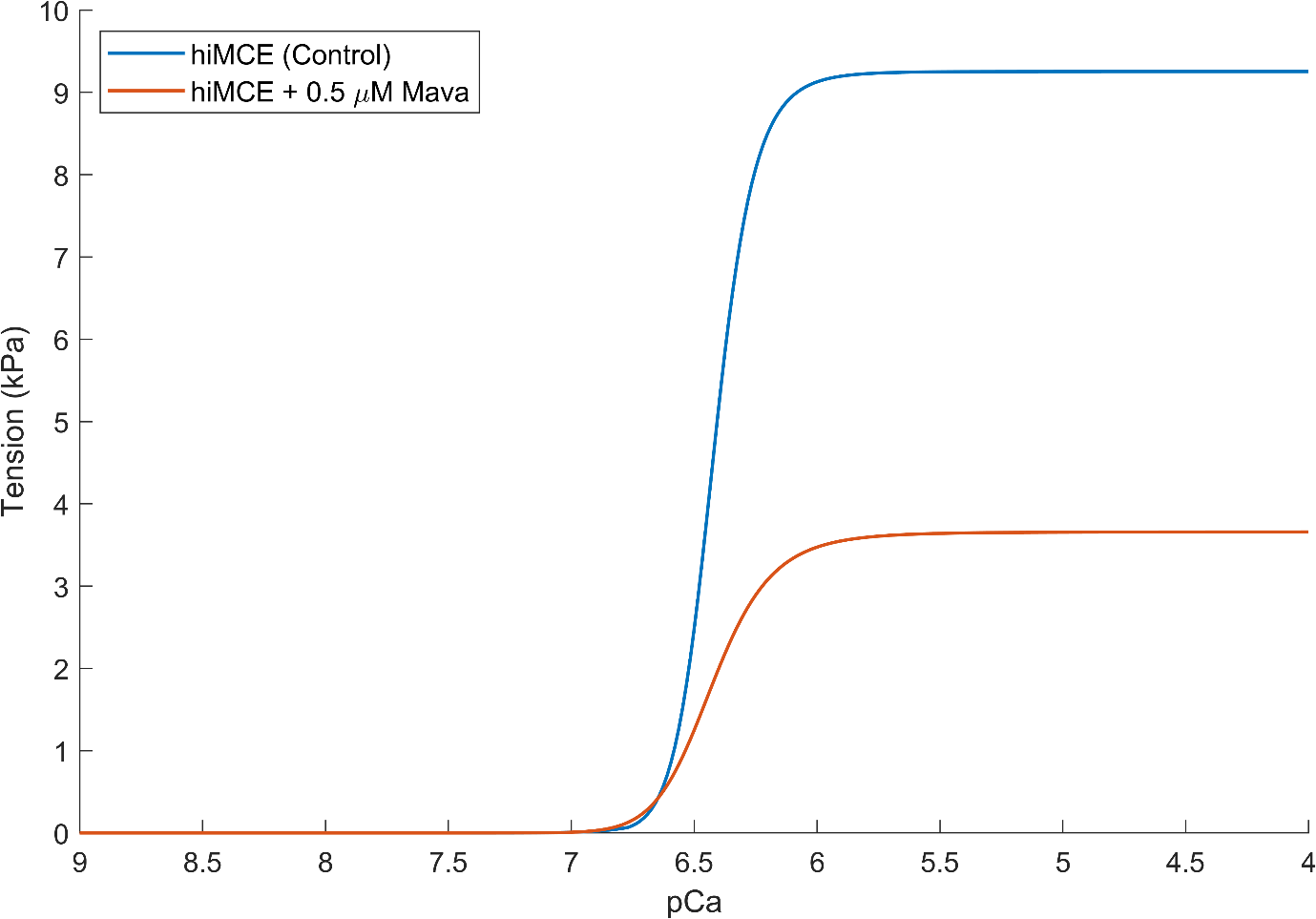


Fig. S1 Tension-Ca^2+^ relationships and the effect of 0.5 µM Mavacamten in isometric conditions.


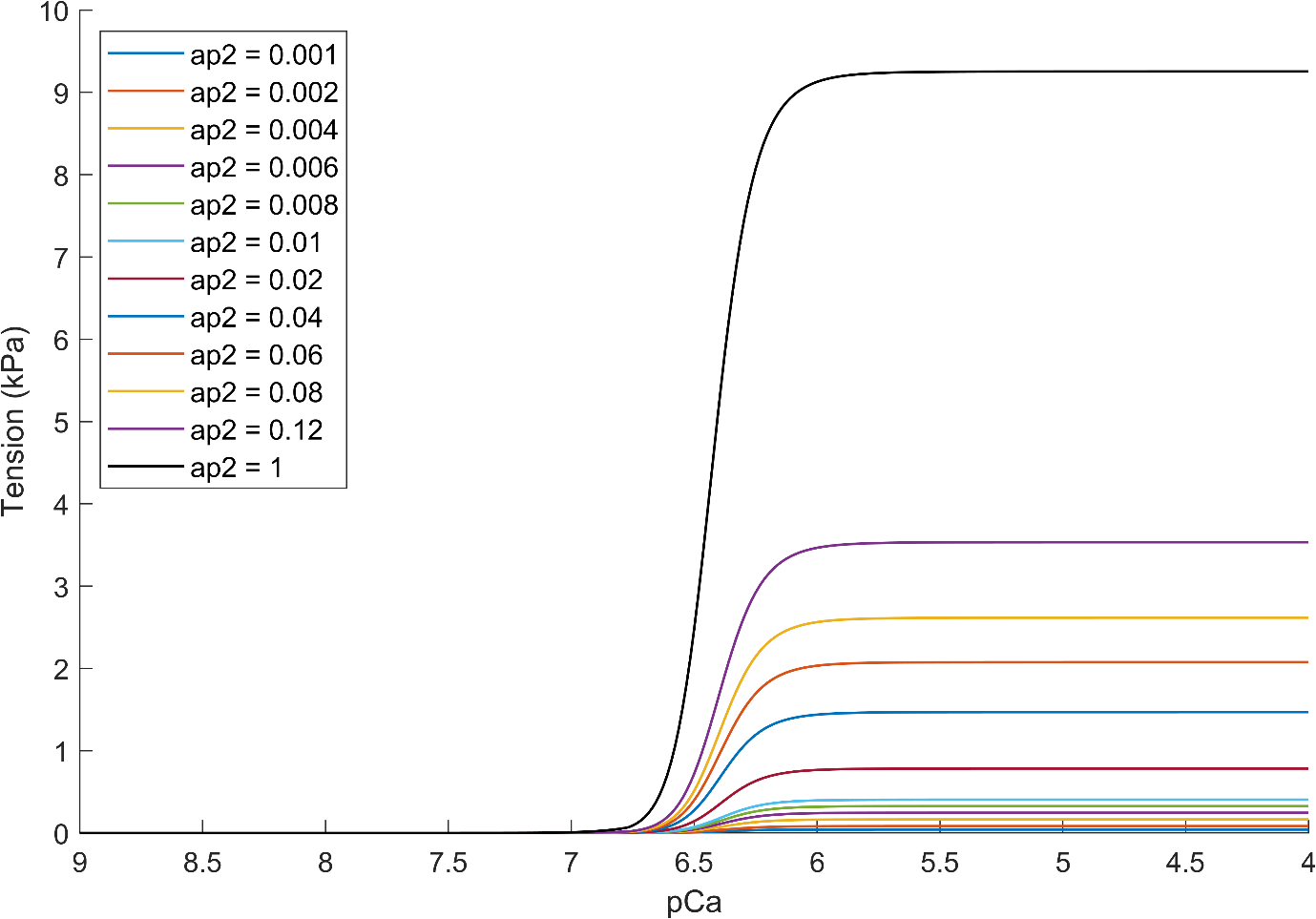


Fig. S2 Tension-pCa relationships in response to ap2 coefficient modulation in isometric condition.


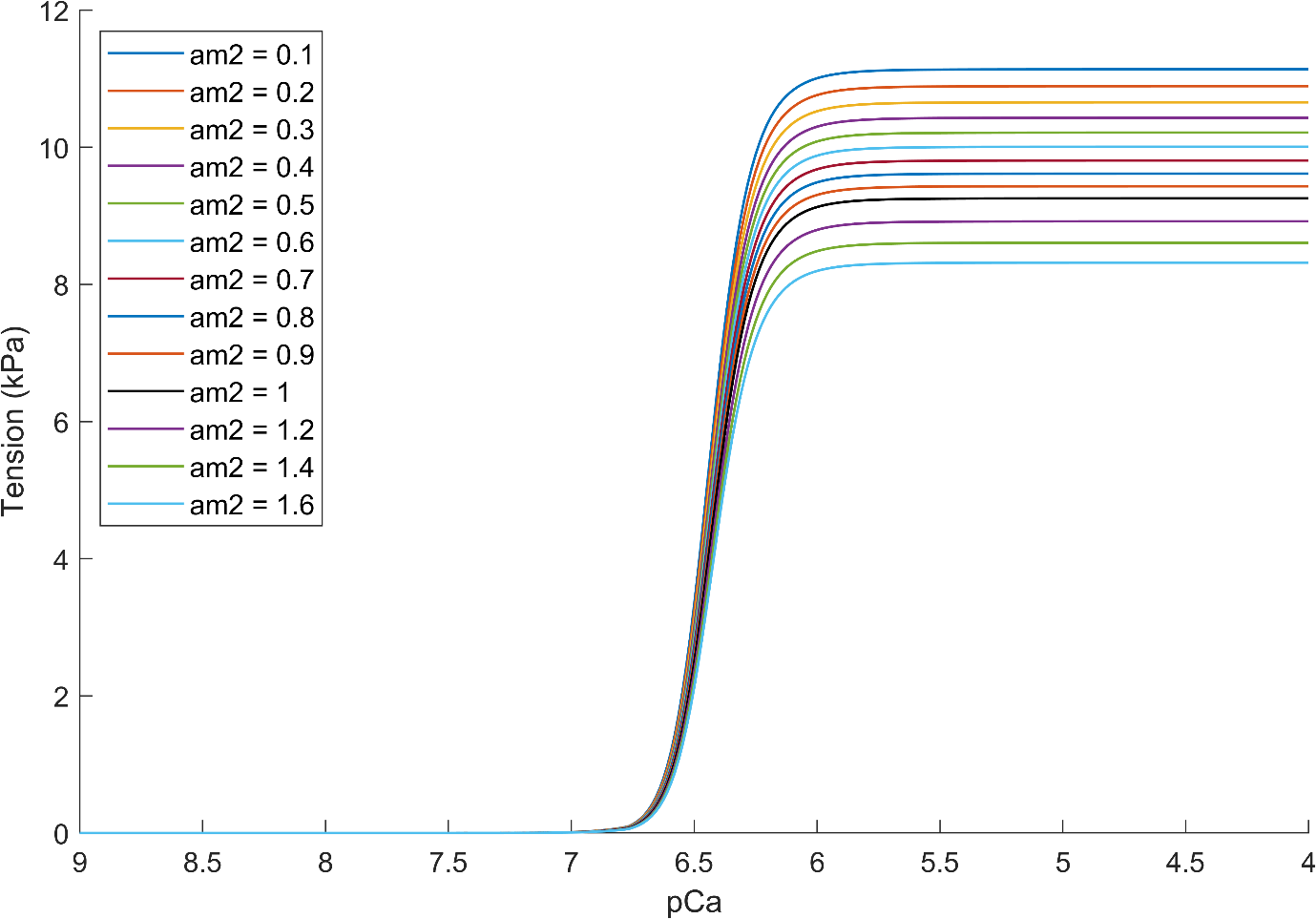


Fig. S3 Tension-pCa relationships in response to am2 coefficient modulation in isometric condition.


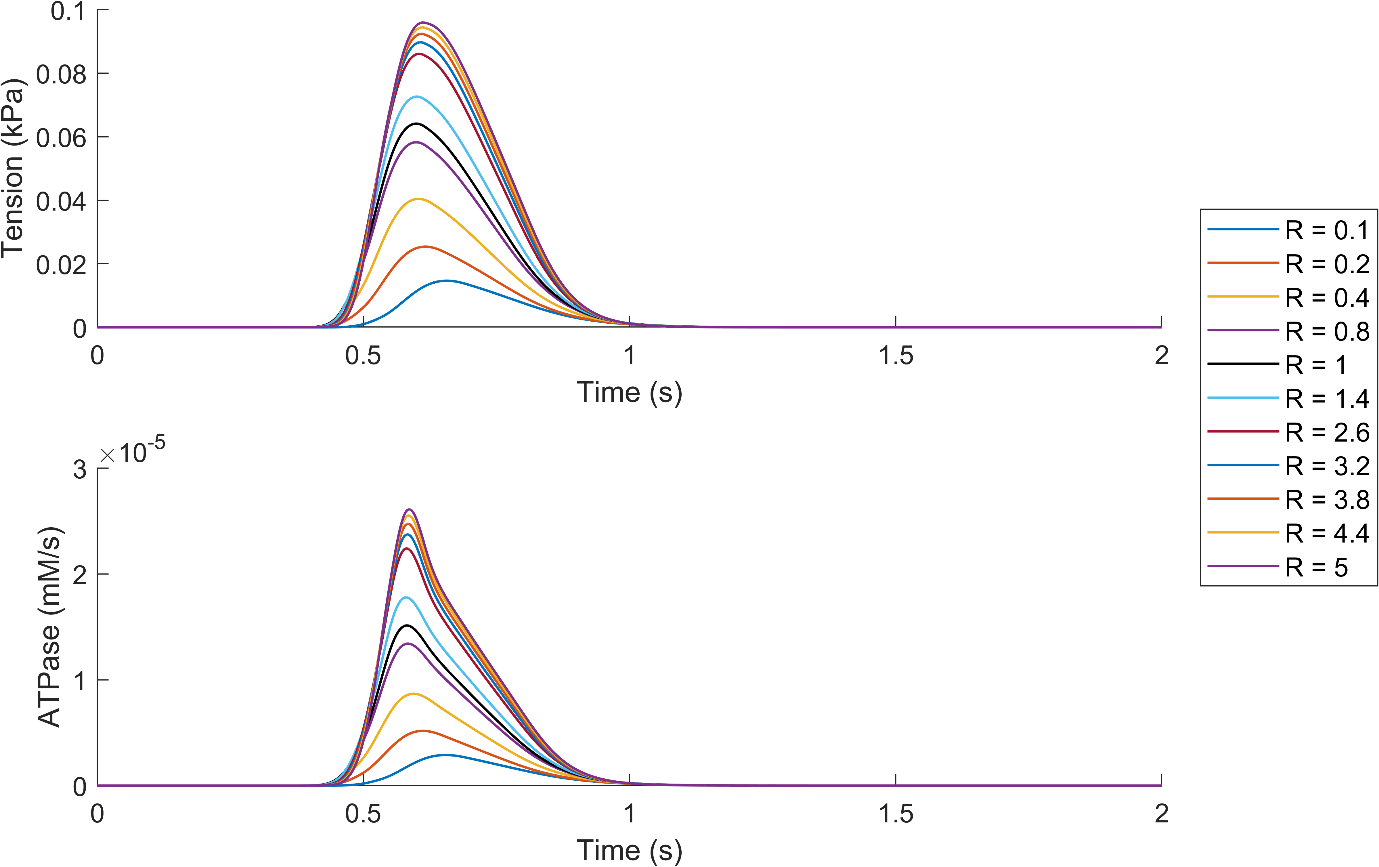


Fig. S4 Active tension (A) and ATPase rate (B) in response to R (=F1=F2) modulation.


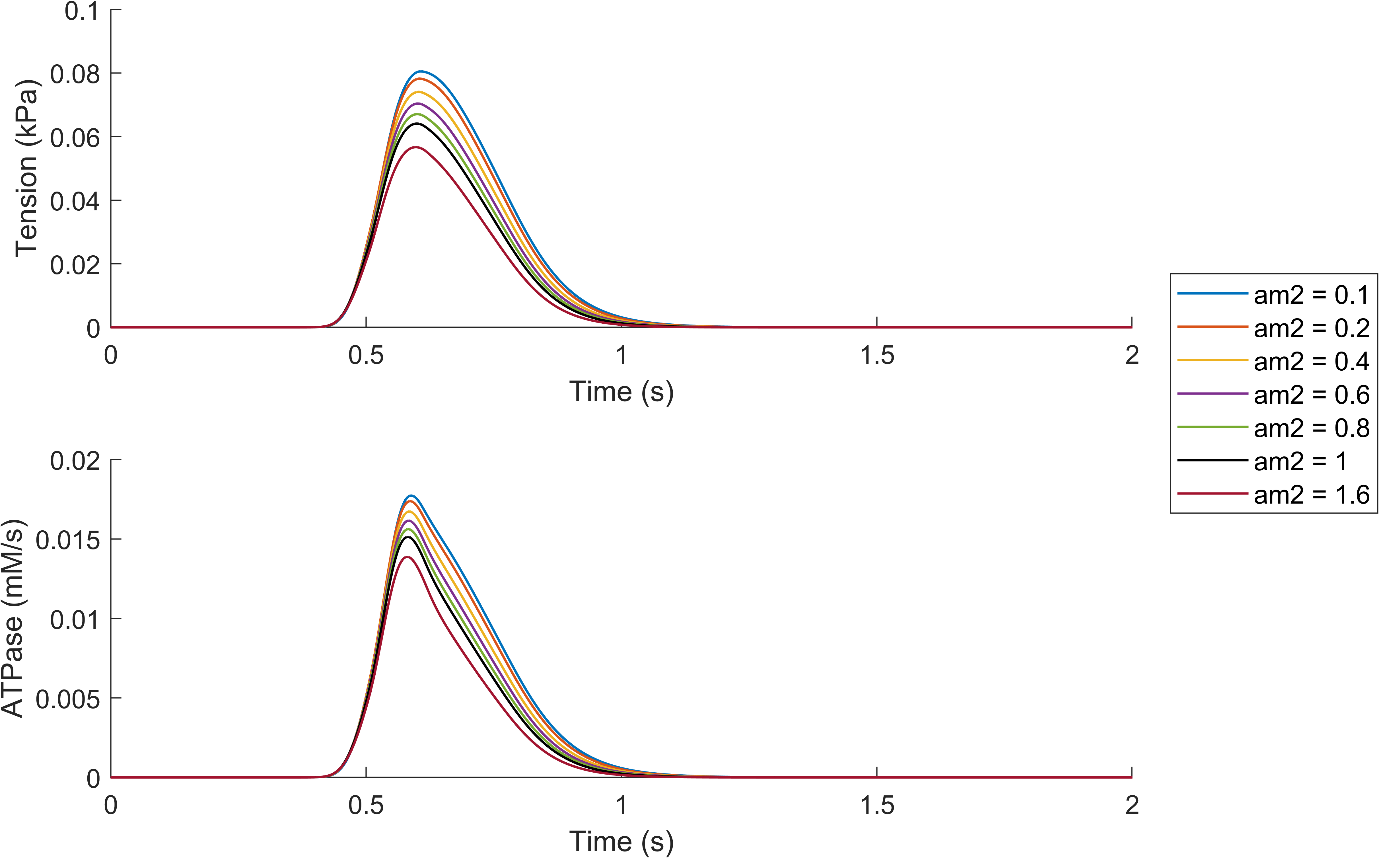


Fig. S5 Active tension (A) and ATPase rate (B) in response to am2 coefficient modulation.

Literature cited

[1] M. Forouzandehmehr, J.T. Koivumäki, J. Hyttinen, M. Paci, A mathematical model of hiPSC cardiomyocytes electromechanics, Physiol. Rep. 9 (2021). doi:10.14814/phy2.15124.

[2] S.G. Campbell, S.N. Flaim, C.H. Leem, A.D. McCulloch, Mechanisms of transmurally varying myocyte electromechanics in an integrated computational model, Philos. Trans. R. Soc. A Math. Phys. Eng. Sci. 366 (2008) 3361–3380. doi:10.1098/rsta.2008.0088.

[3] M.A. Zile, N.A. Trayanova, Rate-dependent force, intracellular calcium, and action potential voltage alternans are modulated by sarcomere length and heart failure induced-remodeling of thin filament regulation in human heart failure: A myocyte modeling study, Prog. Biophys. Mol. Biol. 120 (2016) 270–280. doi:10.1016/j.pbiomolbio.2015.12.012.

[4] M.A. Zile, N.A. Trayanova, Myofilament protein dynamics modulate EAD formation in human hypertrophic cardiomyopathy, Prog. Biophys. Mol. Biol. 130 (2017) 418–428. doi:10.1016/j.pbiomolbio.2017.06.015.

[5] M.A. Zile, N.A. Trayanova, Increased thin filament activation enhances alternans in human chronic atrial fibrillation, Am. J. Physiol. Circ. Physiol. 315 (2018) H1453–H1462. doi:10.1152/ajpheart.00658.2017.
